# Framingham Stroke Risk Profile and poor cognitive function: a population-based study

**DOI:** 10.1186/1471-2377-8-12

**Published:** 2008-04-22

**Authors:** David J Llewellyn, Iain A Lang, Jing Xie, Felicia A Huppert, David Melzer, Kenneth M Langa

**Affiliations:** 1 Department of Public Health and Primary Care, University of Cambridge, Cambridge, UK; 2 Public Health and Epidemiology Group, Peninsula Medical School, Exeter, UK; 3 Department of Psychiatry, University of Cambridge, Cambridge, UK; 4 Department of Internal Medicine, University of Michigan, Michigan, USA; 5 Veterans Affairs Center for Practice Management and Outcomes Research, Michigan, USA; 6 Institute for Social Research, University of Michigan, Michigan, USA

## Abstract

**Background:**

The relationship between stroke risk and cognitive function has not previously been examined in a large community living sample other than the Framingham cohort. The objective of this study was to examine the relationship between 10-year risk for incident stroke and cognitive function in a large population-based sample.

**Methods:**

Participants were 7377 adults aged 50 years and over of the 2002 wave of the English Longitudinal Study of Ageing, a prospective cohort study. A modified version of the Framingham Stroke Risk Profile (incorporating age, sex, systolic blood pressure, antihypertensive medication, diabetes, smoking status, cardiovascular disease, and atrial fibrillation) was used to assess 10-year risk of stroke. Linear regression models were used to determine the cross-sectional relationship of stroke risk to global cognitive function and performance in multiple cognitive domains.

**Results:**

In unadjusted models 10 percentage point increments of 10-year stroke risk were associated with poor global cognitive function (-0.40 SD units, 95% CI -0.43 – -0.38), and lowered performance in all cognitive domains. After statistical adjustment for age, sex, testing interval and other correlates of cognitive function the association with stroke risk was attenuated though remained significant for global cognitive function (-0.06 SD units, 95% CI -0.09 – -0.03), immediate and delayed verbal memory, semantic verbal fluency and processing speed.

**Conclusion:**

In individuals free from a history of stroke or dementia, high subclinical cerebrovascular disease burden was associated with worse cognitive function in multiple domains.

## Background

Risk factors for incident stroke such as smoking status and diabetes predispose older adults to brain atrophy, white matter abnormalities and silent cerebral infarction in addition to clinical stroke, [1-4] and biological aging of the brain is partly attributable to aging of the cerebrovascular circulation and the effects of these vascular changes on the brain.[5,6] Differences in subclinical cerebrovascular pathology may therefore help to explain the marked individual differences in cognitive function and rate of decline observed during late adulthood.[7,8] As Hachinski recently noted, the relationship between subclinical cerebrovascular pathology and cognition has effectively been neglected, and provides a great opportunity for prevention if implemented at the 'brain at risk' stage.[9]

Brady and colleagues[6] observed that the Framingham Stroke Risk Profile (FSRP),[10,11] a previously validated index of cerebrovascular disease burden, predicted 3-year semantic verbal fluency decline. Their analyses incorporated 235 male Veterans Administration patients free of stroke or dementia, and they controlled for age, education, and baseline performance. However, they observed no association between stroke risk and decline in memory (including immediate and delayed verbal recall) or visual-spatial function. They argued that stroke risk factors may therefore have a particularly damaging effect on frontally mediated cognitive functions (e.g. semantic verbal fluency) in comparison with functions mediated by other brain regions (e.g. memory). Their study was limited by the small institutionalized male sample and the lack of adjustment for additional correlates of cognition.

Elias and colleagues discovered significant associations between a modified FSRP and a wide range of cognitive abilities (abstract reasoning, visual-spatial memory, visual organization, attention, visual scanning and tracking) in 1011 men and 1164 women free of dementia and stroke from the Framingham Offspring Study.[12] However, stroke risk was not significantly associated with verbal memory or paired associate learning. They adjusted for age, sex, education, depressed mood, alcohol consumption, BMI, and total serum cholesterol in their full covariate model.  The sample was largely composed of highly educated individuals, and the authors argue that further research is necessary to extend this work to a more representative study population. Recent American Heart Association and the American Stroke Association Stroke Council guidelines also stress the need for further research using the FSRP with groups other than the Framingham cohort.[13] Given that the main evidence linking stroke risk and cognitive function incorporates the same sample used for the development of the FSRP itself, further research is clearly needed to examine whether this association generalizes to an independent and more representative cohort.

We used a modified version of the FSRP to study the relationship between stroke risk and cognitive function. The major risk factors for stroke are combined and weighted to produce a convenient and cost-effective score that predicts the 10-year probability of incident stroke in stroke-free individuals. The FSRP is predictive of brain atrophy,[4] which is in turn associated with an increased subsequent risk of dementia.[14,15] The relationship between the FSRP and cognition has not previously been examined in a large population-based sample other than the Framingham cohort itself. Our objective was to investigate whether stroke risk was associated with cognitive function in a large nationally representative population.

## Methods

### Subjects

Participants were from the 2002 wave of the English Longitudinal Study of Ageing (ELSA) who were born between 1901 and 1952.[16] Following ethical approval from the Multicentre Research and Ethics Committee the ELSA sample was drawn from households participating in the Health Survey for England (HSE), a nationally representative multi-stage stratified random sample of the community living English population. First, 1759 postcode sectors (geographic areas) were selected from the Postcode Address File, stratified by health authority and proportion of households in non-manual socioeconomic groups. Second, households were randomly selected, and a specified number of adults and children were deemed eligible for interview.  Households were included in ELSA if one or more individuals living there was aged 50 or over. 11392 individuals took part in the HSE and ELSA and were eligible for the analysis. The final ELSA sample contained 708 additional respondents who had joined eligible households since HSE, though these individuals were precluded from our analyses. Comparison with census results suggests that the ELSA sample has the same demographic profile as the English community living population.[16]  Information on all FSRP components was collected from a subsample of 7716 individuals. 339 (4%) participants with a history of clinical stroke or dementia were excluded. The remaining 7377 participants formed the sample for our analyses.

### Stroke Risk Profile

The FSRP is a widely used clinical score based on the prediction of 427 stroke events observed over a 10-year follow up period for 2372 men and 3362 women in the Framingham Heart Study.[10,11] The FSRP is based on the following risk factors: age, systolic blood pressure, antihypertensive medication, diabetes, cigarette smoking status, history of cardiovascular disease, atrial fibrillation, and left ventricular hypertrophy as determined by ECG. Two of the FSRP components were determined from the HSE (systolic blood pressure and antihypertensive medication use). Systolic blood pressure (mm Hg) was recorded as the average of three nurse-recorded measurements taken on the right arm with the informant in a seated position after five minutes rest. The remaining FSRP components were assessed at the 2002 wave of ELSA. Diabetes was defined as a previous diagnosis of diabetes or use of a hypoglycaemic agent or insulin. Participants were categorized as current cigarette smokers or not by current cigarette smoking status. Previous diagnoses of cardiovascular disease (defined here as coronary heart disease, congestive heart failure, or peripheral vascular disease) and atrial fibrillation were also incorporated.  We did not incorporate left ventricular hypertrophy in the present analysis as ECG data were not available. Risk of stroke was defined as the 10-year risk or predicted probability of incident stroke expressed as a percentage.[11] Levels of 10-year stroke risk ranged from 3% to 68% for men and 1% to 84% for women.

### Neuropsychological Test Battery

The neuropsychological tests incorporated in the 2002 wave of ELSA to assess cognition are summarized below and described in detail elsewhere.[17] Time orientation was assessed using questions from the Mini-Mental State Examination (MMSE).[18] Immediate and delayed verbal memory were assessed using a 10-word learning task from the Health and Retirement Study (HRS).[19] Ten common words are presented aurally by computer at a rate of one word every 2 seconds. The sound level is adjusted to meet the requirements of each participant. Participants are then asked to recall as many words as possible immediately and again after a short delay during which they complete other cognitive tests. Four different randomly assigned word lists are used, and members of the same household are given different versions.  Prospective memory (or 'remembering to remember') was assessed by asking participants to remember to write their initials in the top left corner of a page of a clipboard when it is handed to them later in the session (closely based on a task incorporated in the MRC Cognitive Function and Ageing Study [MRC CFAS]),[20] and asking participants to remind the interviewer to record the time when the interviewer announces that the cognitive section is finished. Numeracy was assessed using questions relating to simple calculations based on everyday situations, and these items have also been incorporated in the HRS.[19] The semantic verbal fluency task was taken from the CAMCOG,[21] and examines how many animals people are able to name in one minute. The same task has been used in many other studies, including MRC CFAS. Attention and processing speed were assessed using the letter cancellation task from the MRC National Study of Health and Development.[22] Participants are asked to cross out as many of the 65 target letters (P and W) as possible in 1 minute on a page incorporating 780 letters in a grid. The total number of letters searched provides a measure of processing speed. The ratio of correctly identified target letters to all target letters scanned provides a measure of search accuracy. As the scoring of each individual test varies, we standardized test scores to give a mean of 0 and a standard deviation of 1 (z-scores). We obtained a global cognitive function score by standardizing the summed z-scores on all neuropsychological tests.

### Statistical Analysis

Multivariable linear regression models were used to determine the relationship of 10-year stroke risk to cognitive function and performance in multiple cognitive domains. We first examined unadjusted models. To facilitate interpretation we examined 10-year stroke risk (FSRP scores) in 10 percentage point increments. For basic adjustment we controlled for interval in years between blood pressure measurement and cognitive testing, and those elements of the FSRP that are not amenable to intervention (age and sex). In full covariate models we added additional correlates of cognitive function in order to examine whether 10-year stroke risk was independently predictive: education (school leaving age in years), socioeconomic status (based on National Statistics-socioeconomic classification 3 based occupational categories: professional/managerial, intermediate, routine/manual),[23] total net non-housing wealth, BMI (wt/ht^2^), alcohol consumption (g/day), and depressive symptoms (> 3 depressive symptoms on the 8-item Center for Epidemiological Studies Depression Scale [CESD]).[24,25] Analyses of FSRP components were undertaken to further explore their independence as risk factors. We conducted sensitivity analyses to adjust for total serum cholesterol (mmol/L) for a subsample of participants (n = 2746), and examine whether the same pattern of associations was observed for those with and without educational qualifications. Official ELSA weights were used to adjust for the survey design.[16] All statistical levels quoted (*P *values) are two-tailed. Data were analyzed using Stata SE version 9.2.[26]

## Results

The characteristics of the study sample are described in Table 1. Men had a significantly higher 10-year risk of stroke than women, and were significantly younger than women. A greater proportion of men had diabetes and cardiovascular disease than women. Descriptive statistics for the neuropsychological tests are presented in Table 2. Women performed better on time orientation, immediate verbal memory, delayed verbal memory, and processing speed. Conversely men performed better on prospective memory, semantic verbal fluency, and numeracy.

**Table 1:** Characteristics of the Study Population*

Variables	Men (n = 3315)	Women (n = 4062)	*P*†
Framingham Stroke Risk Profile‡, %	10.5 (8.7)	9.5 (11.4)	< 0.001
Stroke risk components			
Mean age, y	64.0 (10.5)	65.6 (12.5)	< 0.001
Mean systolic blood pressure§ mm Hg	141.6 (18.8)	141.6 (22.7)	0.961
Treatment with antihypertensive drugs§ %	29.9	29.2	0.600
History of diabetes, %	8.2	5.4	< 0.001
Current smoker, %	15.2	15.0	0.765
History of cardiovascular disease, %	14.0	9.3	< 0.001
History of atrial fibrillation, %	6.4	5.3	0.060
Additional variables			
Education (leaving age, y), %			< 0.001
< 15	23.1	24.7	
15–17	58.2	59.9	
> 17	18.7	15.5	
Socioeconomic status||, %			< 0.001
Managerial and professional	36.9	23.5	
Intermediate	19.5	28.6	
Routine and manual	43.6	48.0	
Mean total net non-housing wealth, £	96,161.4 (331,157.4)	82,419.7 (294,015.5)	0.034
Alcohol consumption§ %			< 0.001
≥ 30 g/day	72.4	83.7	
0.1–29.9 g/day	20.4	3.1	
None	7.2	13.1	
Mean BMI§ wt/ht^2^	27.7 (4.0)	27.5 (4.8)	0.143
Depressive symptoms (8-item CESD > 3), %	11.1	17.9	< 0.001
Mean total serum cholesterol§ mmol/L	4.6 (2.8)	4.8 (3.2)	0.030

CESD indicates Center for Epidemiological Studies Depression Scale. Values are mean (SD) where appropriate.

*339 persons with a history of stroke or dementia were excluded.

†*P *value for mean difference between men and women.

‡Excluding left ventricular hypertrophy.

§Variable derived from the Health Survey for England (HSE).

||Based on current or former occupation according to National Statistics-socioeconomic classification 3. [23]

**Table 2:** Neuropsychological Test Raw Scores*

		Men (n = 3315)		Women (n = 4062)		
			
Cognitive Domain	Range	Mean	SD	Mean	SD	*P*†
Time orientation	0 – 4	3.67	0.69	3.73	0.61	< 0.001
Immediate verbal memory	0 – 10	5.38	1.76	5.50	1.75	0.002
Delayed verbal memory	0 – 10	3.92	2.08	4.07	2.11	0.001
Prospective memory	0 – 2	1.12	0.80	0.99	0.80	< 0.001
Semantic verbal fluency	0 – 8	19.94	6.39	18.83	6.20	< 0.001
Processing speed	0 – 780	295.26	92.71	317.30	96.11	< 0.001
Attention	0 – 1	0.79	0.14	0.79	0.14	0.957
Numeracy	0 – 6	4.31	1.35	3.58	1.33	< 0.001

*339 persons with a history of stroke or dementia were excluded.

†*P *value for mean difference between men and women.

We used linear regression analyses to determine the relationship between 10-year stroke risk and global cognitive function. In the unadjusted model 10-year stroke risk was clearly associated with cognitive function (*β *= -0.405, 95% CI -0.434 – -0.376, *P *< 0.001). In other words, for every 10 percentage point increase in 10-year stroke risk cognitive function worsens by 0.4 SD units. This association was attenuated after adjustment for testing interval and FSRP risk factors not amenable to intervention (age and sex), though remained significant (*β *= -0.086, 95% CI -0.117 – -0.056, *P *< 0.001). Similarly in the fully adjusted model the association between stroke risk and cognitive function remained significant (*β *= -0.057, 95% CI -0.089 – -0.025, *P *< 0.001). The association between stroke risk and poor cognitive function observed was not simply due to the associations with age, sex and additional variables associated with cognition.

Performance in all individual cognitive domains was significantly associated with stroke risk in unadjusted linear regression models (Table 3). For example, each 10 percentage point increment in 10-year stroke risk was associated with a decrement of 0.355 SD for delayed verbal memory. In the full covariate models stroke risk remained significantly associated with immediate verbal memory, delayed verbal memory, semantic verbal fluency, and processing speed. As the range of values for prospective memory and time orientation were limited to a small number of discrete categories we calculated additional ordered logistic regression models, though this made no difference, and the pattern of results was not sensitive to the choice of model.

**Table 3:** Regression Coefficients Expressing Cognitive Function Decrements (in SD units) for Each 10 Percentage Point Increment in 10-Year Risk of Stroke*

	Unadjusted models			Fully adjusted models†		
		
Cognitive Domain	*β*	(95% CI)	*P*	*β*	(95% CI)	*P*
Time orientation	-0.131	(-0.160 – -0.103)	< 0.001	-0.022	(-0.058 – 0.014)	0.231
Immediate verbal memory	-0.332	(-0.360 – -0.305)	< 0.001	-0.037	(-0.073 – -0.001)	0.044
Delayed verbal memory	-0.355	(-0.381 – -0.329)	< 0.001	-0.064	(-0.097 – -0.032)	< 0.001
Prospective memory	-0.244	(-0.267 – -0.221)	< 0.001	-0.026	(-0.059 – 0.007)	0.127
Semantic verbal fluency	-0.276	(-0.300 – -0.252)	< 0.001	-0.032	(-0.063 – -0.002)	0.038
Processing speed	-0.185	(-0.212 – -0.157)	< 0.001	-0.045	(-0.084 – -0.007)	0.022
Attention	-0.167	(-0.194 – -0.141)	< 0.001	-0.015	(-0.053 – 0.024)	0.449
Numeracy	-0.181	(-0.206 – -0.157)	< 0.001	-0.007	(-0.040 – 0.026)	0.683

Pooled data are shown as sex-specific analyses gave similar results. Persons with a history of stroke or dementia were excluded.

*Modified Framingham Stroke Risk Profile excluding left ventricular hypertrophy.

†Adjusted for age, sex, testing interval, education, socioeconomic status, total net non-housing wealth, BMI, alcohol consumption, and depressive symptoms.

In order to further investigate which individual risk factors were driving the results, each of the FSRP components was related to global cognitive function. In an unadjusted linear regression model all individual risk factors were inversely and significantly related to cognitive function (range, *β *= -0.152, *P *< 0.001 to *β *= -0.011, *P *= 0.03), with the exception of sex which was not significantly associated (*β *= 0.002, *P *= 0.904), and atrial fibrillation which was positively and significantly associated (*β *= 0.024, *P *= 0.003). In an adjusted model (also incorporating education, testing interval, socioeconomic status, total net non-housing wealth, BMI, alcohol consumption and depressive symptoms) age, diabetes, smoking, cardiovascular disease and sex (males higher) were inversely and significantly associated with cognitive function (range, *β *= -0.118, *P *< 0.001 to *β *= -0.031, *P *< 0.01). However, neither systolic blood pressure (*β *= -0.005, *P *= 0.286) nor atrial fibrillation (*β *= 0.015, *P *= 0.071) were significantly associated with cognitive function in the adjusted model.

Finally, sensitivity analyses to examine the robustness of our results showed that the same association between stroke risk and cognitive function was observed when total cholesterol was also adjusted for in a subsample of participants. Analysing those with and without educational qualifications separately also showed the same pattern of results. The association between stroke risk and cognitive function was not therefore limited to highly educated participants.

## Discussion

### Summary

In a large population-based cohort of English men and women with no prior history of dementia or stroke, we found a 10 percentage point increase in 10-year stroke risk was associated with a large decrement in global cognitive function (-0.40 SD units), and poor performance all cognitive domains. The association between stroke risk and cognitive function was not simply a reflection of age and sex as risk factors, lending support to the notion that the aggressive treatment of cerebrovascular risk factors may be effective in preserving cognitive health. In full covariate models (adjusted for age, sex, testing interval, socioeconomic status, total net non-housing wealth, BMI, alcohol consumption, and depressive symptoms) stroke risk remained significantly associated with global cognitive function, immediate and delayed verbal memory, semantic verbal fluency and processing speed.

### Critique of methods

The strengths of our study include the large population-based sample and the wide range of neuropsychological tests incorporated. We used survey weights to adjust for the complex sampling design, and comparison with census results suggests that ELSA has the same demographic profile as the English community living population.[16] Practice or learning effects were not an issue as we examined the first administration of these tests. We were also able to adjust for a wide range of potential confounders. As many of the risk factors for poor cognition are also risk factors for stroke,[9] this may have misleadingly attenuated the relationship between stroke risk and cognitive function. However, the inverse association observed remained significant even in the full covariate model, suggesting that stroke risk is robustly and independently associated with levels of cognitive function.

Our analyses were restricted to community living individuals. However, dementia is highly prevalent in institutional care in England,[27] and as we excluded individuals with a history of dementia many of these individuals would have been excluded. The possibility of residual confounding and the cross-sectional design preclude us from making definitive conclusions about causality, and the reliance on self-reported diagnosed medical conditions (including stroke and dementia) is also a limitation. There was evidence for a ceiling effect with the time orientation questions, which may have attenuated their association with the FSRP. As ECG data were not available we did not incorporate left ventricular hypertrophy in the present analyses. However, the prevalence of left ventricular hypertrophy is very low,[28] it is given little weighting in the FSRP,[10,11] and the association between left ventricular mass and cognition is rendered non-significant after adjustment for cardiovascular risk factors and cardiovascular disease.[29]

### Results in comparison with literature

FSRP scores observed in the present analyses were higher than those observed by Elias and colleagues,[12] as in comparison the ELSA cohort was slightly older, had higher mean systolic blood pressure, and a higher proportion of current smokers. Our results are consistent with their results suggesting that after adjustment for age, sex, education and other correlates of cognition 10-year stroke risk is associated with poor cognition in multiple domains. These findings run contrary to a small longitudinal study of institutionalized males.[6] suggesting that the association between cognition and stroke risk is confined to semantic verbal fluency (or frontally mediated forms of cognition). It appears that stroke risk is associated with cognition in multiple cognitive domains, even after adjustment for a wide range of potential confounders.

Our findings are also consistent with a broader body of research suggesting that individual risk factors for stroke are associated with cognitive function.[9] While the neuropsychological tests incorporated in our analyses differ from those used by Elias and colleagues, both studies included measures of processing speed that were associated with stroke risk in full covariate models. While we observed an association with verbal memory, Elias and colleagues did not. This may reflect differences in the verbal memory tasks incorporated, as we incorporated a list learning task and Elias and colleagues included a paragraph recall task. Age, sex, education, BMI, alcohol consumption, and depressive symptoms were adjusted for in the full covariate models of both studies. Elias and colleagues adjusted for total serum cholesterol, while we adjusted for this in a sensitivity analysis with a subsample of individuals. Elias and colleagues' sample was largely comprised of highly educated individuals, whereas the ELSA sample is more diverse and shares the same sociodemographic profile as the community living population. Our study therefore extends our understanding of the relationship between stroke risk and cognitive function by demonstrating that it is not restricted to highly educated individuals or the cohort used to develop the FSRP itself.  This study is also the first to incorporate additional adjustment for socioeconomic status and wealth, which are well established correlates of cognitive function.[30]

Subclinical cerebrovascular disease may provide an important link between the major risk factors for stroke and cognition. Brain atrophy, white matter abnormalities and silent cerebral infarction are possible mechanisms underlying the association between stroke risk and cognitive function. [1-4] For example, the FSRP has been shown to predict brain atrophy,[4] which is in turn associated with an increased subsequent risk of dementia.[14,15] In the population-based Rotterdam Scan Study white matter hyperintensities, generalized brain atrophy, and brain infarcts on MRI were associated with steeper decline in processing speed and executive function during 5.2 years mean follow-up.[31] After excluding participants with an incident stroke several associations were no longer significant, suggesting that stroke risk is predictive of decline in processing speed and executive function. Similarly, in a 3-year prospective study of 554 subjects,[32] periventricular white matter hyperintensity volume at baseline predicted longitudinal decline in processing speed.

## Conclusion

In summary, this is the first study to examine the relationship between stroke risk and cognitive function in a large population-based sample in a cohort other that that used to develop the FSRP itself. Our findings are important as they suggest that risk of stroke is robustly associated with cognitive function in stroke- and dementia-free individuals, likely reflecting differences in subclinical cerebrovascular pathology. Stroke risk remained significantly associated with cognitive function in multiple domains even after adjustment for a wide range of factors correlated with cognition.

This association was observed in individuals with and without educational qualifications. The FSRP may provide a convenient and cost-effective way to identify vulnerable older adults at the 'brain at risk' stage,[9] and further research is needed to establish whether the targeted aggressive treatment of cerebrovascular risk factors is an effective preventative strategy for cognitive decline.

## Competing interests

The authors declare that they have no competing interests.

## Authors' contributions

DL, IAL, DM and KML conceived of the study. DL acquired the data and DL and JX conducted the statistical analysis. DL, IAL, FAH, DM and KML participated in the management, analysis, interpretation of data, and drafting of the manuscript. All have critically revised the manuscript for important intellectual content and seen and approved the final version.

## Pre-publication history

The pre-publication history for this paper can be accessed here:


